# A neural joint model for entity and relation extraction from biomedical text

**DOI:** 10.1186/s12859-017-1609-9

**Published:** 2017-03-31

**Authors:** Fei Li, Meishan Zhang, Guohong Fu, Donghong Ji

**Affiliations:** 1 School of Computer, Wuhan University, Bayi Road, Wuhan, China; 2 School of Computer Science and Technology, Heilongjiang University, Xuefu Road, Harbin, China

**Keywords:** Biomedical text, Entity recognition, Relation extraction, Neural network, Joint model

## Abstract

**Background:**

Extracting biomedical entities and their relations from text has important applications on biomedical research. Previous work primarily utilized feature-based pipeline models to process this task. Many efforts need to be made on feature engineering when feature-based models are employed. Moreover, pipeline models may suffer error propagation and are not able to utilize the interactions between subtasks. Therefore, we propose a neural joint model to extract biomedical entities as well as their relations simultaneously, and it can alleviate the problems above.

**Results:**

Our model was evaluated on two tasks, i.e., the task of extracting adverse drug events between drug and disease entities, and the task of extracting resident relations between bacteria and location entities. Compared with the state-of-the-art systems in these tasks, our model improved the F1 scores of the first task by 5.1% in entity recognition and 8.0% in relation extraction, and that of the second task by 9.2% in relation extraction.

**Conclusions:**

The proposed model achieves competitive performances with less work on feature engineering. We demonstrate that the model based on neural networks is effective for biomedical entity and relation extraction. In addition, parameter sharing is an alternative method for neural models to jointly process this task. Our work can facilitate the research on biomedical text mining.

## Background

Automatically extracting entities and their relations from biomedical text has attracted much research attention in biomedical text mining community due to its important applications on knowledge acquisition and ontology construction [1]. Recently, various related tasks have been proposed, such as protein-protein interaction detection (PPI) [2], drug-drug interaction detection (DDI) [3], adverse drug event extraction (ADE) [4] and the bacteria biotope task (BB) [5].

Taking the ADE task for example, the objective of this task is to recognize mentions of drug and disease entities, and extract possible ADE relations between them. Given a sentence “A woman who was treated for **thyrotoxicosis**_*disease*_ with **methimazole**_*drug*_ developed **agranulocytosis**_*disease*_.”, the outputs will be three entity mentions and an ADE relation {**methimazole**_*drug*_, **agranulocytosis**_*disease*_} _*ADE*_.

Entity and relation extraction is a standard task in text mining or natural language processing (NLP). Most of previous work used two-step pipeline models to perform this task. First, entity mentions in a given sentence are recognized using the technologies of named entity recognition (NER). NER is usually casted as a sequence labeling problem solved by conditional random fields (CRFs) [6]. Second, each entity pair is examined to decide whether they have task-specific relations using classification models such as support vector machines (SVMs) [7]. In the biomedical community, pipeline models are also frequently used for this task [8–14].

Such pipeline models suffer two main problems. First, the errors generated in the NER step may propagate to the step of relation classification. For instance, if a drug or disease entity mention is incorrectly recognized, the extraction of its related ADEs will be incorrect. Second, the interactions between two subtasks in the two steps are not able to be utilized, while these interactions may help the subtasks. For instance, given a sentence “The tire maker still employs 1400” [15], although it may be difficult to recognize “1400” as a person entity, the word “employs” indicates an employment-organization relation which must involve a person entity. Therefore, such relation may help the model to recognize “1400” correctly.

Due to the aforementioned disadvantages of pipeline models, joint models, which process entity recognition and relation classification simultaneously, have been proposed. Joint models process two subtasks simultaneously, so they can alleviate the problem of error propagation. On the other hand, some model parameters are shared by the submodels of entity recognition and relation classification in joint models, so these parameters help the models capture the interactions between two subtasks. Roth and Yih [16] proposed a joint inference framework based on integer linear programming to extract entities and relations. Li and Ji [15] exploited a single transition-based model to accomplish entity recognition and relation classification simultaneously. Kordjamshidi et al. [17] proposed a structured learning model to extract biomedical entities and their relationships. However, these feature-based approaches require much feature engineering and they also suffer feature sparsity problem, since the combined feature space of a joint task is significantly larger than those of its subtasks.

Recently, deep learning with neural networks has received increasing research attention in the artificial intelligence area [18, 19], as well as the text mining and NLP areas [20, 21]. Compared with other models, deep neural networks adopt low-dimensional dense embeddings to denote features such as words or part-of-speech (POS) tags, which can effectively settle the feature sparsity problem. In addition, deep neural networks demand less feature engineering, since they can learn features from training data automatically. Ma and Hovy [22] and Lample et al. [23] exploited similar frameworks by combining recurrent neural networks (RNNs) with CRFs and obtained the best results on several benchmark NER datasets. For relation classification, there are two state-of-the-art methods using deep neural networks, namely RNNs [24] and convolutional neural networks (CNNs) [25]. They used RNNs or CNNs to learn relation representations along the words between two target entities or along the words on the shortest dependency path (SDP) of two target entities. Miwa and Bansal [26] proposed an end-to-end relation extraction model and obtained competitive performances in several datasets. However, there is less related work in biomedical entity and relation extraction using deep neural networks. Li et al. [27] and Mehryary et al. [28] used similar approaches with [24, 25], but they only focused on relation classification with given entities. Li et al. [29] exploited a transition-based feed-forward neural network to jointly extract drug-disease entity mentions and their ADE relations. Jiang et al. [30] proposed two independent neural models for DDI and gene mention tagging tasks, respectively.

In this paper, we follow the novel line of work on deep neural networks and propose a neural joint model to extract biomedical entities and their relations. First, our model uses CNNs to encode character information of words into their character-level representations. Second, character-level representations, word embeddings and POS embeddings are fed into a bi-directional (Bi) long short-term memory (LSTM) [31] based RNN to learn the representations of entities and their contexts in a sentence. These representations are used to recognize biomedical entities. Third, another Bi-LSTM-RNN learns relation representations of two target entities along their SDP. These representations are used to classify their relations. The second Bi-LSTM-RNN is stacked on the first one, i.e., the output vectors of LSTM units in the first Bi-LSTM-RNN are used as the input vectors of LSTM units in the second one. The parameters of LSTM units in the first Bi-LSTM-RNN are shared by both networks, so they are jointly affected by entity recognition and relation classification tasks during training.

Our neural joint model was evaluated for extracting biomedical entities and their relations on two tasks, namely ADE [4] and BB [5]. Comparing with the state-of-the-art model [29] for the ADE task, our model improved the precision and recall of drug-disease entity recognition by 3.2 and 7.1%, and ADE relation extraction by 3.5 and 12.9%, respectively. Comparing with the best system [14] for the BB task, our model boosted the precision and recall of resident relation extraction by 30.5 and 0.8%, respectively. Experimental results showed that our neural joint model could obtain competitive performances with less feature engineering. In addition, our model could obtain better performances than pipeline models by sharing parameters between the submodels. We demonstrate that deep neural networks are also effective for biomedical entity and relation extraction. Therefore, our model is able to facilitate the research on biomedical text mining.

## Methods

### CNN for character-level representations

**Fig. 1:**
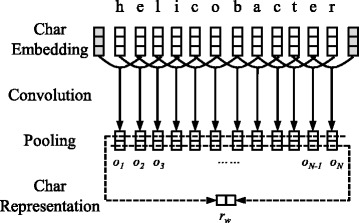
The CNN for extracting character-level representations. A rectangular grid indicates a vector and a square indicates one dimension of this vector, so character embeddings or representations can be denoted as n-dimensional vectors. Shading rectangular grids indicate special padding vectors

Character-level features have been demonstrated to be effective for neural NER models. For example, the suffix “bacter” is a strong feature to indicate a bacteria entity such as “campylobacter” or “helicobacter”. Following previous work [22, 23], CNNs are used to extract morphological information (like the prefix or suffix of a word) from characters of words. Figure 1 shows the process of extracting character information from a word and encoding them into a character-level vector representation.

Given a word *w*={*c*_1_,*c*_2_,…,*c*_*N*_}, *c*_*i*_ denotes its *i*-th character and *emb*(*c*_*i*_) denotes the embedding of this character. To use morphological information, the embeddings of continuous characters in a window size *C* are concatenated as the final representation $r_{c_{i}}$ of *c*_*i*_. For example, if $C=1, r_{c_{i}} = [\!emb(c_{i-1}), emb(c_{i}), emb(c_{i+1})]$, where “[]” denotes the vector concatenation operation. Then the convolutional kernel of CNN needs *N* times of convolutions for all the characters in this word and for each convolution *i*, the kernel output *o*_*i*_ is computed by

1 $$ o_{i} = tanh\left(W_{1} r_{c_{i}} + b_{1}\right),   $$

where *W*_1_ and *b*_1_ are the parameter matrix and bias vector that are learned, and *tanh* denotes the hyperbolic tangent activation function. To generate the character-level representation *r*_*w*_ of this word *w*, max-pooling operations are applied to all kernel outputs *o*_1_,*o*_2_,…,*o*_*N*_. The *k*-th dimension of *r*_*w*_ is computed by

2 $$ r_{w_{k}} = \max_{1 \leqslant i \leqslant N} o_{i_{k}}.   $$

### Bi-LSTM-RNN for biomedical entity recognition

**Fig. 2:**
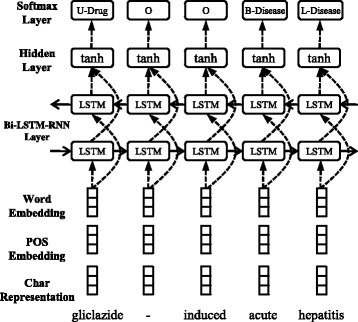
The Bi-LSTM-RNN for biomedical entity recognition. *Rectangular grids* indicate vectors of feature embeddings or representations. At the *bottom*, three kinds of vectors are concatenated and fed into LSTMs. *Dashed arrow lines* denote bottom-up computations along the network framework and *solid arrow lines* denote left-to-right computations along the sentence

Following state-of-the-art neural models [22, 23, 26], biomedical entity recognition is casted as a sequence labeling problem. For example, if the standard label scheme *BILOU* is utilized in the ADE task, which includes two entity types namely *Drug* and *Disease*, entity labels can be designed as follows. *B-Drug*/*B-Disease*, *I-Drug*/*I-Disease* and *L-Drug*/*L-Disease* denote the beginning, following and last words of *Drug*/*Disease* entities, respectively. *U-Drug* or *U-Disease* denotes the single word of *Drug* or *Disease* entities. *O* denotes that the word does not belong to any type of entities. For example, given a sentence “gliclazide-induced acute hepatitis”, Fig. 2 shows the process of labeling each word of this sentence by our Bi-LSTM-RNN model.

Given a sentence $\phantom {\dot {i}\!}w_{1}/p_{1}/r_{w_{1}}, w_{2}/p_{2}/r_{w_{2}}, \ldots, w_{N}/p_{N}/r_{w_{N}}$, where *w*_*i*_ denotes the *i*-th word, *p*_*i*_ denotes the POS tag of *w*_*i*_, and $\phantom {\dot {i}\!}r_{w_{i}}$ denotes the character-level representation of *w*_*i*_. For the *i*-th step of sequence labeling, the Bi-LSTM-RNN layer takes the concatenation of the word embedding, POS tag embedding and character-level representation of *w*_*i*_ as inputs, given by

3 $$  t_{i}=\left[emb\left(w_{i}\right), emb\left(p_{i}\right), r_{w_{i}}\right].  $$

Based on *t*={*t*_1_,*t*_2_,…,*t*_*N*_}, a LSTM unit in the left-to-right direction associates each of them with a hidden state $\overrightarrow {h}_{i}$, so *t* corresponds to $\overrightarrow {h}=\{\overrightarrow {h}_{1}, \overrightarrow {h}_{2}, \ldots, \overrightarrow {h}_{N}\}$. Here $\overrightarrow {h}_{i}$ does not only capture the information in the current step, but also that in the previous steps. To capture the information in the following steps, we also add a counterpart $\overleftarrow {h}_{i}$ of $\overrightarrow {h}_{i}$ in the reverse direction, so *t* also corresponds to $\overleftarrow {h}=\{\overleftarrow {h}_{1}, \overleftarrow {h}_{2}, \ldots, \overleftarrow {h}_{N}\}$. In the hidden layer, $\overrightarrow {h}_{i}$ and $\overleftarrow {h}_{i}$ are selected as one input source in the *i*-th step. Moreover, the last entity label *l* $^{e}_{i-1}$ is also selected as another input source to consider label dependence (e.g., the label *I-Drug* should not follow the label *O*). This is not shown in Fig. 2 for conciseness. The final inputs and outputs of the *i*-th step in the hidden layer are given by

4 $$ h^{e}_{i} = tanh\left(W_{2} \left[\overrightarrow{h}_{i}, \overleftarrow{h}_{i}, emb\left(l^{e}_{i-1}\right)\right] + b_{2}\right),   $$

where $h^{e}_{i}$ denotes the output vector of the hidden layer, *W*_2_ and *b*_2_ denote the parameter matrix and bias vector that are learned.

Finally, the softmax output layer calculates the probabilities *y* ^*e*^ of all entity labels *L* ^*e*^, given by

5 $$ y^{e} = softmax\left(W_{3} h^{e}_{i} + b_{3}\right),   $$

where the *k*-th label with the maximum probability $y^{e}_{k}$ is selected as the label of the *i*-th word.

### Bi-LSTM-RNN for relation classification

**Fig. 3:**
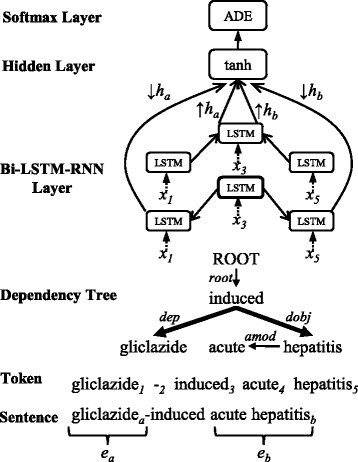
The Bi-LSTM-RNN for relation classification. The input sentence is tokenized before it is analyzed by a dependency parser. Tokens are indexed by Arabic numerals. Basic (a.k.a, projective) dependency style is utilized to build a tree. The bold lines in the tree denote the shortest dependency path (SDP) between “gliclazide” and “hepatitis” with their lowest common ancestor “induced”. *x*_*i*_ indicates the input vector of a LSTM unit as shown in Eq. 6 and *i* corresponds to the index of a token. In the Bi-LSTM-RNN layer, solid arrow lines denote bottom-up and top-down computations along the SDP in the dependency tree. *↑* *h*_*a*_, *↑* *h*_*b*_, *↓* *h*_*a*_, *↓* *h*_*b*_ are listed in Eq. 8

Once entity recognition is finished, our model starts relation classification to determine whether a task-specific relation exists between all possible entity pairs. Prior work has demonstrated the effectiveness of SDPs in the dependency trees for relation classification [24, 26]. The words along SDPs concentrate on most relevant information while diminishing less relevant noise. Following these studies, we use the Bi-LSTM-RNN to model relation representations between two target entities along their SDP. For example, given a sentence “gliclazide-induced acute hepatitis”, Fig. 3 shows the process of classifying ADE relations by our Bi-LSTM-RNN.

Given an entity pair *e*_*a*_ (e.g., gliclazide) and *e*_*b*_ (e.g., acute hepatitis) in a sentence, the last words *a* (e.g., gliclazide) and *b* (e.g., hepatitis) of these entities are used to build the SDP between them. The SDP can be formally represented by {*a,a*_1_,…,*a*_*m*_,*c,b*_*n*_,…,*b*_1_,*b*} (e.g., {gliclazide, induced, hepatitis}), where *c* denotes their lowest common ancestor in the dependency tree (e.g., induced). *a*_1_,…,*a*_*m*_ denote the words occurring between *a* and *c* on the SDP, and *b*_1_,…,*b*_*n*_ denote the words occurring between *b* and *c*. The SDP can be divided into two parts: {*a,a*_1_,…,*a*_*m*_,*c*} (e.g., {gliclazide, induced}) and {*b,b*_1_,…,*b*_*n*_,*c*} (e.g., {hepatitis, induced}) are bottom-up sequences; {*c,a*_*m*_,…,*a*_1_,*a*} (e.g., {induced, gliclazide}) and {*c,b*_*n*_,…,*b*_1_,*b*} (e.g., {induced, hepatitis}) are top-down sequences. We extract features from both kinds of sequences by the Bi-LSTM-RNN. The input of each LSTM unit is a concatenation of three parts, given by

6 $$ x_{i} = \left[\overrightarrow{h}_{i}, \overleftarrow{h}_{i}, emb(d_{i})\right],  $$

where *emb*(*d*_*i*_) denotes the embedding of dependency type *d*_*i*_ between the word *w*_*i*_ and its governor in the dependency tree. $\overrightarrow {h}_{i}$ and $\overleftarrow {h}_{i}$ correspond to the word *w*_*i*_ and they are identical to those notations mentioned in Eq. 4. Since $\overrightarrow {h}_{i}$ and $\overleftarrow {h}_{i}$ are used as the inputs of these LSTM units, the Bi-LSTM-RNN for relation classification is stacked on the Bi-LSTM-RNN for entity recognition. Therefore, two Bi-LSTM-RNNs in our joint model share partial parameters and these parameters can be tuned during jointly training, which assists our joint model to capture the interactions between two subtasks. Miwa and Bansal [26] also demonstrated the effectiveness of such method for neural models.

The last LSTM outputs computed along bottom-up sequences {*a,a*_1_,…,*a*_*m*_,*c*} and {*b,b*_1_,…,*b*_*n*_,*c*} are denoted as *↑* *h*_*a*_ and *↑* *h*_*b*_. The last LSTM outputs computed along top-down sequences {*c,a*_*m*_,…,*a*_1_,*a*} and {*c,b*_*n*_,…,*b*_1_,*b*} are denoted as *↓* *h*_*a*_ and *↓* *h*_*b*_.

In the hidden layer, *↑* *h*_*a*_,*↑* *h*_*b*_,*↓* *h*_*a*_ and *↓* *h*_*b*_ are selected as one input source, and the entity representations *r*_*a*_ and *r*_*b*_ are used as another input source, computed by

7 $$  \begin{aligned} r_{a} &= \frac{1}{|K_{a}|}\sum_{k \in K_{a}} \left[\overrightarrow{h}_{k}, \overleftarrow{h}_{k}\right],\\ r_{b} &= \frac{1}{|K_{b}|}\sum_{k \in K_{b}} \left[\overrightarrow{h}_{k}, \overleftarrow{h}_{k}\right], \end{aligned}  $$

where *K*_*a*_ and *K*_*b*_ denote the index sets of the words in two entities, and $\overrightarrow {h}_{k}$ and $\overleftarrow {h}_{k}$ are identical to those notations in Eq. 4. Entity representations are used to compensate information losses, since the SDP are built according to the last words of two target entities. For conciseness, this part is not shown in Fig. 3.

Finally, all vector representations of two input sources are concatenated and then computed in the hidden layer to generate the outputs *h* ^*r*^, given by

8 $$ h^{r} = tanh\left(W_{4} \left[\uparrow{h}_{a}, \uparrow{h}_{b}, \downarrow{h}_{a}, \downarrow{h}_{b}, r_{a}, r_{b}\right] + b_{4}\right).   $$

A softmax layer calculates the probabilities *y* ^*r*^ of all relation labels *L* ^*r*^, given by

9 $$ y^{r} = softmax\left(W_{5} h^{r} + b_{5}\right),   $$

where the *k*-th label with the maximum probability $y^{r}_{k}$ is selected as the relation type of two target entities *e*_*a*_ and *e*_*b*_.

### Training

Both submodels of our joint model employ the same training algorithm and AdaGrad [32] is employed to control the update step. We describe their training in one section for conciseness.

Online learning is exploited to train model parameters. Given a sentence with gold-standard entities and relations, we generate some training examples for entity recognition and relation classification submodels. When each example is sent to its corresponding submodel, the cross-entropy loss for this example is computed and gradients are back-propagated to each layer of the submodel for updating parameters. Therefore, we can consider two submodels are trained alternately. Moreover, since the parameters of LSTM units in the entity recognition submodel are shared by two submodels, the loss of each example can propagate to these parameters. Therefore, they are affected by both entity recognition and relation classification tasks.

Formally, assuming that the gold-standard label and its predicted probability are *l* and *prob*_*l*_, the loss for each example is calculated via - log*prob*_*l*_. If all losses are accumulated with a L_2_ regularization term, the final objective is given by

10 $$  L(\theta)\ =\ -\sum\limits_{i} \log{prob_{l}}\ +\ \frac{\lambda}{2}\ \lVert\ \theta\ \rVert^{2}_{2},  $$

where *θ* denotes all model parameters, and *λ* is the regularization parameter.

### Data

We carried out experiments on two tasks, namely adverse drug event extraction (ADE) [4] and the bacteria biotope task (BB) [5].

The ADE task aims to extract two kinds of entities (drugs and diseases) and relations about which drug is associated with which disease (ADEs). Its dataset is published in the form of independent sentences that come from 1644 PubMed abstracts. Sentences in the dataset are divided into two categories, namely 6821 sentences in which at least one drug/disease entity pair has the ADE relation (i.e., ADE sentences), and 16695 sentences in which no drug/disease entity pair has the ADE relation (i.e., non-ADE sentences). Biocurators only annotated drug/disease entities (i.e., the arguments of ADE relations) in the ADE sentences, so there are no annotated entities in the non-ADE sentences. Following previous work [29], only ADE sentences were used in our experiments since we need to evaluate the performances of both entity recognition and relation extraction. Similar to prior work [12, 29], 120 relations with nested gold annotations were removed (e.g., “lithium intoxication”, where “lithium” is related to “lithium intoxication”).

The BB task aims to extract bacteria-related knowledge from PubMed abstracts. We focus on the *BB-event+ner* subtask, which consists of two parts, namely recognizing bacteria, habitat and geographical entity mentions, and extracting *Lives_In* relations between bacteria entities and their locations (either habitat or geographical entities). The training, development and test set of the *BB-event+ner* subtask include 71, 36 and 54 documents, which contain 1158, 736, 1049 entities and 327, 223, 314 relations, respectively. The statistics of the final data used in our experiments are shown in Table 1.

**Table 1:** Statistics of the ADE and BB data used in our experiments

ADE		BB	
Sentences	6821	Documents	161
Entities	10666	Entities	2943
Relations	6686	Relations	864

### Evaluation metrics

Standard precision (*P*), recall (*R*), *F1* were used as evaluation metrics of entity and relation extraction, computed by

11 $$ \begin{aligned} P &= \frac{TP}{TP + FP},\\ R &= \frac{TP}{TP + FN},\\ F {1} &= \frac{2 \times P \times R}{P + R},\\ \end{aligned}   $$

where a recognized entity mention was counted as true-positive (*TP*) if its boundary and type matched those of a gold entity mention. An extracted relation was counted as *TP* if its relation type was correct, and the boundaries and types of its related entities matched those of the entities in a gold relation. A recognized entity or extracted relation was counted as false-positive (*FP*) if it did not match the corresponding conditions mentioned above. The number of false-negative (*FN*) instances was computed by counting the gold entities or relations that had not been identified by our model.

Since there were no official development set in the ADE task, we evaluated our model using 10-fold cross-validation, where 10% of the data were used as the development set, 10% were used as the test set and the remaining were used as the training set. Then the final results were displayed as macro-averaged scores.

For the BB task, we used *P*, *R* and *F1* to evaluate our model on the development set. The final results on the test set were given by the official evaluation service [5], which showed only the overall performance of relation extraction in *P*, *R* and *F1*.

### Hyper-parameter settings

Some of hyper-parameter values were tuned according to the development set and others were chosen empirically following prior work [22, 26] since it is infeasible to perform full search for all hyper-parameters. Their final values are shown in Table 2. For conciseness, the dimensions of model parameter matrices *W*_1_,*W*_2_,*W*_3_,*W*_4_,*W*_5_ and bias vectors *b*_1_,*b*_2_,*b*_3_,*b*_4_,*b*_5_ are not shown since they can be easily deduced from this table. Their values were randomly initialized with a uniform distribution.

**Table 2:** Hyper-parameter settings

Type	Hyper-parameter
Training	*α*=0.03,*λ*=10^−8^
Embedding	*dim*(*emb*(*w*_*i*_))=200
	$dim(emb(p_{i}), emb(d_{i})~\text {or}~emb(l^{e}_{i}))=25$
CNN	*dim*(*emb*(*c*))=25,*C*=3
	*dim*(*r*_*w*_)=25
Bi-LSTM-RNN (Entity)	$dim(\overrightarrow {h}_{i})$ or $dim(\overleftarrow {h}_{i})=100$
	$dim(h^{e}_{i})=100$
Bi-LSTM-RNN (Relation)	*dim*(*↑* *h*_*a*_,*↑* *h*_*b*_,*↓* *h*_*a*_ or *↓* *h*_*b*_)=100
	*dim*(*h* ^*r*^)=100

*dim* denotes vector dimensions and *emb* denotes feature embeddings

The initial AdaGrad learning rate *α* and regularization parameter *λ* were set to 0.03 and 10 ^−8^, respectively. The dimension of word embeddings was set to 200 and those of other feature embeddings were set to 25. We used pre-trained biomedical word embeddings [33] to initial our word embeddings and other kinds of embeddings were randomly initialized in the range (-0.01, 0.01). All the embeddings were tuned during training except word embeddings.

For CNN, the character window size *C* was set to 3, so the dimension of convolutional kernel inputs *r*_*c*_ can be computed as (2 ×3+1) ×25=175. For Bi-LSTM-RNN in entity recognition, we set the dimensions of LSTM hidden states $\overrightarrow {h}_{i}$ or $\overleftarrow {h}_{i}$, and the hidden layer $h^{e}_{i}$ to 100. For Bi-LSTM-RNN in relation classification, we set the dimensions of LSTM hidden states *↑* *h*_*a*_,*↑* *h*_*b*_,*↓* *h*_*a*_ or *↓* *h*_*b*_, and the hidden layer *h* ^*r*^ to 100. The dimensions of entity representations *r*_*a*_ and *r*_*b*_ can be computed as 200.

### Preprocessing

Given a document, we used some heuristic rules to split it into sentences and then tokenized these sentences into words. Tokenization was performed using not only whitespaces but also punctuations, since we might not find the node for an entity (e.g., “gliclazide”) in the dependency tree if it was not separated from a piece of text (e.g., “gliclazide-induced”). All the words were transformed into their lowercase forms and numbers were replaced by zeroes. The version 3.4 of Stanford CoreNLP toolkit [34] was used for POS tagging and dependency parsing. To ensure dependency structures as trees, we employed basic (a.k.a., projective) dependencies. In particular, the discontinuous and nested entities were removed, in order to fit our model.

## Results

### Result comparisons with other work

Table 3 shows the results of prior work that processed the ADE task. Kang et al. [12] utilized a knowledge-based pipeline method, namely recognizing entities via an off-the-shelf tool, and extracting ADEs via the UMLS Metathesaurus and Semantic Network [35]. As shown in Table 3, their method obtained the imbalanced precision and recall. One likely reason is that their method did not distinguish between ADE relations and drug-disease treatment relations due to the limitations of manually designed rules and knowledge bases, so this strategy led to a high recall but a low precision. By contrast, our neural joint model achieved more balanced precisions and recalls without the assistance of knowledge bases. In addition, the recall of relation extraction is comparable with that of their method.

**Table 3:** Result (%) comparisons with other work in the ADE task

Method	Entity recognition			Relation extraction		
	P	R	F1	P	R	F1
Kang [12]	—	—	—	42.1	76.3	54.3
Li [29]	79.5	79.6	79.5	64.0	62.9	63.4
Our model	82.7	86.7	84.6	67.5	75.8	71.4

Li et al. [29] used a feed-forward neural network to jointly extract drug-disease entities and ADE relations. For drug-disease entity recognition, our model improved the precision, recall and F1 by 3.2, 7.1 and 5.1%, respectively. For ADE relation extraction, the precision, recall and F1 was improved by 3.5, 12.9 and 8.0%, respectively. Their method used knowledge bases such as WordNet [36] and CTD [37] to help improving performances. Moreover, they manually designed global features to capture the interactions of entity recognition and relation extraction. By contrast, our model obtained much better results without using any knowledge base and captured the interactions automatically.

Table 4 shows the results of related work that processed the BB task. LIMSI [14] achieved the best F1 in the official evaluation. It leveraged a pipeline framework using CRF to recognize mentions of bacteria and locations, and SVM to extract *Lives_In* relations between two entity mentions. UTS [5] also employed a pipeline framework that relied on two independent SVMs to perform entity recognition and relation classification, respectively. As shown in Table 4, they suffered either low precisions or recalls. Our neural joint model outperformed their methods without using knowledge bases provided by the task organizers. In addition, neural features reduced the work of feature engineering in CRF or SVM.

**Table 4:** Result (%) comparisons with other work in relation extraction of the BB task

	LIMSI	UTS	Our model
Precision	19.3	33.1	49.8
Recall	19.1	13.3	19.9
F1	19.2	19.0	28.4
F1(Habitat)	18.6	17.4	29.2
F1(Geographical)	28.3	35.0	20.5
F1(Intra-sentence)	28.6	23.4	35.1

All the methods in the BB task achieved lower recalls than precisions, which might be caused by two reasons. The first reason is that there is much disagreement among annotators on whether to annotate an entity mention or relation as a gold answer based on the official statistics [5] shown in Table 5. This implies that it is a challenging task to extract *Lives_In* relations from PubMed abstracts, even for professional annotators. The second reason is that there are 27% inter-sentence relations (i.e., the argument entities of a relation occurring in different sentences) based on the official statistics of BB task, so the methods restricted to extract intra-sentence relations (i.e., the argument entities of a relation occurring in the same sentence) will suffer low recalls. Nevertheless, the extraction of inter-sentence relations is still a very challenging problem in the text mining or NLP area, which is not taken into account for the moment in this paper.

**Table 5:** The inter-annotator agreement (%) of entity mentions and *Lives_In* relations [5]

	P	R	F1
Entity Mentions	95.5	62.1	75.3
*Lives_In* Relations	95.2	31.1	46.8

### Feature contributions

The experiments were carried out on the development set to explore the contributions of different features. For entity recognition, our features consist of words, characters, POS tags and entity labels. For relation extraction, our features consist of words, dependency types, entity representations. In feature contribution experiments, we took the model using word features as the baseline, and added only one kind of other features at a time.

In Table 6, entity labels were most useful in the ADE task, improving the precision and recall by 2.4 and 1.9%, respectively. While in the BB task, POS tags contributed the most, improving the precision and recall by 2.3 and 4.1%, respectively. The effectiveness of character features was moderate, improving the *F1* by 0.3 and 1.3%.

**Table 6:** Feature contribution experiments for entity recognition

Features	ADE			BB		
	P	R	F1	P	R	F1
Word	80.1	83.6	81.8	67.1	56.7	61.4
+char	80.2	84.0	82.1	66.4,	59.4	62.7
+pos	80.5	84.7	82.5	69.4	60.8	64.8
+label	82.5	85.5	84.0	66.1	59.5	62.6
All	82.4	86.4	84.3	68.0	63.4	65.6

Here “+” means only that feature is added. “char”, “pos” and “label” denote character, POS tag and entity label features, respectively

In Table 7, by adding entity representations, our model achieved the biggest improvements in *F1*, by 1.0% in the ADE task and 3.0% in the BB task. While dependency type features contributed the most for the precision in the BB task.

**Table 7:** Feature contribution experiments for relation extraction

Features	ADE			BB		
	P	R	F1	P	R	F1
Word	62.7	69.9	66.1	34.5	20.4	25.6
+dep	63.3	71.0	66.9	42.0	19.9	27.0
+entity	63.4	71.2	67.1	34.1	24.7	28.6
All	67.3	75.7	71.3	42.7	25.2	31.7

Here “+” means only that feature is added.“dep” and “entity” denote dependency type and entity representation features, respectively

Based on our experiments, the contributions of these features are not consistent in different tasks, which is reasonable due to the characters of these tasks and their datasets.

## Discussion

### Comparisons of joint and pipeline models

Since our model uses parameter sharing to joint two Bi-LSTM-RNN networks, it is necessary to evaluate the effectiveness of such method. To this end, a pipeline model was built without parameter sharing and compared with the joint model.

The pipeline model was built by replacing $\overrightarrow {h}_{i}$ and $\overleftarrow {h}_{i}$ in Eq. 6 with word embeddings *emb*(*w*_*i*_). Therefore, the connections between two Bi-LSTM-RNNs were cut off and they became independent submodels. To be fair, both the pipeline and joint models used only word embedding features.

As shown in Table 8, the performance differences between the pipeline and joint models are slight in the ADE task. While in the BB task, the performance of the joint model is much better than that of the pipeline model, and the F1 scores of the joint model increase by 2.8 and 4.2% in entity recognition and relation classification, respectively. Miwa and Bansal [26] performed similar experiments in other datasets and the performance differences varied between 0.8–1.1%.

**Table 8:** Performance comparisons of joint and pipeline models

Task	Method	Entity recognition			Relation extraction		
		P	R	F1	P	R	F1
ADE	Pipeline	79.6	83.5	81.5	62.5	69.9	66.0
	Joint	80.1	83.6	81.8	62.7	69.9	66.1
BB	Pipeline	67.2	52.0	58.6	26.6	17.7	21.2
	Joint	67.1	56.7	61.4	34.5	20.4	25.6

In general, we believe that parameter sharing between the subtasks of a joint model is effective since these parameters are influenced by correlated subtasks and they can help a joint model capturing the interactions of these subtasks. Nevertheless, such strategy may have few effects on improving performances for a specific task, so the characters of a task also need to be considered.

### Error analysis

The errors were divided into two parts, namely *FP* and *FN*. For entity recognition, both *FP* and *FN* errors can be divided into two types: The boundary of an entity is incorrectly recognized and the type of an entity is incorrectly recognized. For relation extraction, *FP* errors contain two types: the entity mentions of a relation are incorrect (either boundaries or types), and entity mentions are correct but their relation is incorrectly predicted. *FN* errors consist of two types: First, at least one entity mention of a relation has not been recognized, leading to losing this relation; Second, both entity mentions of a relation have been recognized, but the model does not determine that they have such relation.

The statistics of error analysis was performed on the development sets of two datasets. As shown in Table 9, boundary identification seems to be much more difficult than type identification in biomedical entity recognition. The errors of boundary identification account for more than 90% of total errors in both tasks. This may be rational due to the following reasons: First, there are only several entity types in the ADE (*drug*/*disease*) and BB (*bacteria*/emphhabitat/*geographical*) tasks, so it is easier for the model to identify entity types; Second, the characters of biomedical entities are more obvious than those of the entities in the common area, which helps the model to identify their types. For example, a bacteria entity “helicobacter” or drug entity “gliclazide” is much less ambiguous than an organization entity “bank”, since “bank” has another meaning “riverside”; Third, the boundary of a biomedical entity is more difficult to be identified, since it may include a number of words to express an integrated biomedical concept, such as a disease entity “bilateral lower leg edema” or habitat entity “monocyte-like THP-1 cells”.

**Table 9:** Error analysis of entity recognition

Task	Error type		%
ADE	FP	Incorrect boundaries	55.3
		Incorrect types	1.3
	FN	Incorrect boundaries	42.1
		Incorrect types	1.3
	Total		100
BB	FP	Incorrect boundaries	37.1
		Incorrect types	3.6
	FN	Incorrect boundaries	55.7
		Incorrect types	3.6
	Total		100

In Table 10, the percentage of the first type of *FP* errors is much higher than that of the second one in both tasks (55.7% vs. 3.1% and 22.7% vs. 15.2%), which implies the importance of entity recognition for relation extraction. The proportion of the second type of *FP* errors in the BB task is larger than that in the ADE task (15.2% vs. 3.1%), which demonstrates the relations in the BB task are more difficult to be predicted.

**Table 10:** Error analysis of relation extraction

Task	Error type		%
ADE	FP	Entities incorrectly recognized	55.7
		Entities correct, relations wrong	3.1
	FN	Entities not found	40.7
		Entities found, relations not found	0.5
	Total		100
BB	FP	Entities incorrectly recognized	22.7
		Entities correct, relations wrong	15.2
	FN	Entities not found	43.7
		Entities found, relations not found	18.4
	Total		100

In addition, the first type of *FN* errors accounts for nearly 50% of total errors in both tasks, which indicates that missing entities is the main reason of missing relations. Therefore, one way to alleviate this problem is to build a high-quality entity recognition model in order to reduce errors propagating to the subsequent step of relation extraction. Another alternative way is to use joint models to alleviate such error propagation. By contrast, the distribution of the second type of *FN* errors shows obvious differences between two tasks. In the ADE task, such errors account for 0.5%, while in the BB task, they account for 18.4%. The reasons for this may be because we only used ADE sentences, which contain at least one ADE relation, as our dataset in the ADE task, since the entities in non-ADE sentences were not annotated. The relation expression in ADE sentences may be apparent so they are easier for the model to determine. In contrast, we used all sentences in the BB task, which increases the difficulty of relation extraction. Furthermore, the relations in the ADE task were annotated in the sentence level, while ones in the BB task were annotated in the document level, so inter-sentence relations were lost.

To further demonstrate our observations from error analysis, we performed additional experiments to compare our model with two relation extraction methods that are based on co-occurrence entities inside one sentence and gold entity mentions. As shown in Table 11, co-occurrence and gold-mention based methods achieved pretty high performances (>95% in F1) in the ADE task, which demonstrates the errors of our model mainly come from entity recognition. Therefore, the low error rates of the second FP (Entities correct, relations wrong: 3.1%) and FN (Entities found, relations not found: 0.5%) in Table 10 are explainable. Achieving high performances when entities are given is mainly due to the annotation method of ADE corpus: if drug and disease entities have no ADE relations in a sentence, entities will not be annotated in that sentence either; therefore, if entities are given, ADE relations are almost determined. By contrast, the submodel of relation classification in our model also contributed a number of errors in the BB task, since co-occurrence and gold-mention based methods achieved modest performances when entities were given. It also explains the high error rates of the second FP (Entities correct, relations wrong: 15.2%) and FN (Entities found, relations not found: 18.4%) in Table 10.

**Table 11:** Comparisons with the methods based on co-occurrence entities inside one sentence and gold entity mentions

Task	Method	Relation Extraction		
		P	R	F1
ADE	Co-occurrence	97.3	100	98.6
	Gold mentions	97.5	99.9	98.7
	Our model	67.3	75.7	71.3
BB	Co-occurrence	34.9	72.5	47.1
	Gold mentions	58.7	43.6	50.0
	Our model	42.7	25.2	31.7

### Limitations of our model

The main limitation of our model is that it is not able to extract inter-sentence relations, which is a much more challenging task since it requires discourse-level language understanding and coreference resolution technologies. Some prior work has explored the methods for inter-sentence relation extraction [38, 39] or event extraction [40]. In future work, our main objective is to alleviate this limitation.

## Conclusions

In this paper, we explore a neural joint model to extract biomedical entities and their relations. Our model utilizes the advantages of several state-of-the-art neural models for entity recognition or relation classification in text mining and NLP. Experimental results on two related tasks showed that our model outperformed the best systems in those tasks. We find that deep neural networks can achieve competitive performances with less work on feature engineering and less dependence on external resources such as knowledge bases. In addition, parameter sharing is an effective method for neural models to jointly process several correlated tasks. We believe that our work can facilitate the research on biomedical text mining, especially for biomedical entity and relation extraction. Whether our model is effective for other biomedical entity-relation-extraction tasks remains to be investigated.

## Abbreviations

ADE: Adverse drug event extraction
BB: Bacteria biotope task
Bi: Bi-directional
CNNs: Convolutional neural networks
CRFs: Conditional random fields
DDI: Drug-drug interaction detection
FN: False-negative
FP: False-positive
LSTM: Long short-term memory
NER: Named entity recognition
NLP: Natural language processing
P: Precision
POS: Part-of-speech
PPI: Protein-protein interaction detection
R: Recall
RNNs: Recurrent neural networks
SDP: Shortest dependency path
SVMs: Support vector machines
TP: True-positive
